# Trade and Deforestation Predict Rat Lungworm Disease, an Invasive-Driven Zoonosis, at Global and Regional Scales

**DOI:** 10.3389/fpubh.2021.680986

**Published:** 2021-09-09

**Authors:** Luz A. de Wit, Taylor H. Ricketts

**Affiliations:** ^1^RubensteinSchool of Environment and Natural Resources, University of Vermont, Burlington, VT, United States; ^2^Gund Institute for Environment, University of Vermont, Burlington, VT, United States

**Keywords:** *Angiostrongylus cantonensis*, environmental degradation, land-use policy, invasive species management, rodent, biosecurity

## Abstract

The introduction of non-native species and deforestation are both important drivers of environmental change that can also facilitate the geographic spread of zoonotic pathogens and increase disease risk in humans. With ongoing trends in globalization and land-use conversions, introduced species and deforestation are ever more likely to pose threats to human health. Here, we used rat lungworm disease, an emerging zoonotic disease caused by *Angiostrongylus cantonensis* and maintained by invasive rats and snails, to explore how these two forms of environmental change can impact zoonotic disease risk. We used logistic regressions to examine the role of global trade in the introduction of *A. cantonensis* at a country level and used model estimates to predict the probability of introduction as a function of trade. We then used hurdle-based regression models to examine the association between deforestation and rat lungworm disease in two regions where *A. cantonensis* is already established: Hawaii and Thailand. At the global scale, we found the trade of horticultural products to be an important driver in the spread of *A. cantonensis* and that the majority of countries at high risk of future *A. cantonensis* introduction are islands. At country scales, we found deforestation to increase the per-capita risk of *A. cantonensis* exposure in Hawaii and Thailand. Our study provides a preliminary view of the associations between species introductions, deforestation, and risk of *A. cantonensis* exposure in people. Better understanding how these two widespread and overlapping forms of environmental change affect human health can inform international biosecurity protocols, invasive species management, and land-use policies.

## Introduction

Species introductions and deforestation are important sources of environmental degradation and biodiversity loss globally (1, 2). These drivers can also lead to the geographic spread of zoonotic pathogens and increase the risk of zoonotic exposure in humans (3, 4). In particular, when non-native species are introduced into new habitats, they can become part of local enzootic or zoonotic disease dynamics or can introduce zoonotic pathogens into new regions (5). Deforestation can increase disease risk in humans because it can result in ecosystem changes that favor reservoirs of zoonotic pathogens, and because deforestation is closely associated with human presence and activities, creating opportunities for contact with zoonotic reservoirs (6–8). Deforestation and species introductions are both continuing global problems that are still on the rise (9, 10), with non-native species likely being introduced into landscapes degraded by deforestation. Thus, understanding the impacts of both drivers of environmental change on zoonotic disease risk will be important for informing public health and land-use policies, to reduce the health burden from emerging zoonotic diseases.

The successful establishment of introduced zoonotic reservoirs largely depends on the number of introduced individuals and frequency of introduction events (propagule pressure) (1, 11). Global trade and travel networks remove natural barriers to species' dispersal and can provide a constant source of propagule pressure for several species (11). Trade and travel are thought to have played a key role in facilitating the introduction of several zoonotic pathogens across the world, such as West Nile virus into North America, possibly through the introduction of infected mosquitoes or birds (12). Other factors intrinsic to the introduced species and ecosystem may further increase the likelihood of establishment and invasion. For example, species with high population growth rates and dietary generalism are more likely to become invasive, particularly if introduced to climatically suitable ecosystems and to communities that have been disassembled (e.g., lack or few competitors and predators) (11, 13).

Deforestation can increase the risk of zoonotic pathogen transmission through multiple mechanisms such as modifying zoonotic reservoirs' habitat and resource availability, changing community composition, and creating opportunities for contact between humans and reservoirs when humans live near forest edges (7, 14). For example, changes in land use that result in changes in water availability such as dam constructions and agricultural irrigation schemes are known to create suitable habitats for *Biomphalaria* spp. snails and *Cullex tarsalis* mosquitoes, increasing the incidence of schistosomiasis and West Nile virus disease, respectively (15, 16). Agricultural and urban land-use types can also provide nutrient subsidies for zoonotic reservoirs in the form of mineral agricultural runoff or human organic waste (17–19). Likewise, deforestation is a leading cause of community disassembly (20), affecting the presence and abundance of natural enemies (i.e., predators and competitors), and allowing certain reservoir hosts to thrive (21). Finally, evidence suggests that human-modified landscapes tend to favor taxa, such as rodents and bats, that have ecological and life-history traits associated with zoonotic reservoirs (22).

While the combined impacts of introduced species and deforestation have been studied in the context of native species and ecosystem processes [e.g., (21, 23, 24)], very few studies have focused on the combined impacts of these two drivers on human health. Here, we explore how introduced species and deforestation can impact global patterns of disease emergence and regional patterns of disease risk. We focus on neuroangiostrongyliasis (rat lungworm disease), an emerging snail-borne disease that is largely maintained by non-native invasive taxa.

Rat lungworm disease is caused by the parasitic nematode *Angiostrongylus cantonensis* and is largely maintained by rats (*Rattus* spp.) and snails and slugs (hereafter referred to as snails) (25). Humans become infected after deliberately or accidentally consuming infected raw snails or paratenic hosts that cannot support the parasite's developmental cycle (e.g., freshwater shrimp, frogs, monitor lizards, flatworms, and centipedes) (25). Infection can result in inflammation of the central nervous system, potentially leading to coma or death (26). Many snail species can serve as intermediate hosts for *A. cantonensis*, yet several widely introduced and highly invasive snails such as giant African snails (*Lissachatina fulica*), apple snails (*Pomacea* spp.), and semi-slugs (*Parmarion cf. martensi*) tend to be frequently infected and carry high parasite loads (27–29). Many of these non-native snails are commonly found in home and community gardens, agricultural plantations, and wetland ecosystems (30–33). Rats are also highly invasive and important commensals of humans (34, 35), and while human exposure to *A. cantonensis* is limited to contact with snails or paratenic hosts, the presence of rats is necessary for completion of the parasite's life cycle (25).

*Angiostrongylus cantonenisis* was first reported in southeastern China in 1935, and both the parasite and disease have since been reported in several regions of the world, including several countries in Africa, Southeast Asia, Oceania, and the Caribbean, several South Pacific islands, Brazil, Ecuador, and several states in the United States (36). The global spread of the parasite has most likely been facilitated by the introduction of infected rats, which has been these species' most common form of dispersal, or through the introduction of infected snails, which can be accidentally or purposefully introduced through the snail and horticulture trade (33, 35, 37). Notably, islands represent a large proportion of the countries where *A. cantonenisis* has been reported (36, 38), a pattern that could be partly explained by many islands' economic dependence on global trade (39–41).

Given the recent range expansion of *A. cantonensis* and its association with introduced invasive species, we focus here on two questions: (1) Does global trade predict the presence of *A. cantonensis* at a country level? (2) In regions where *A. cantonensis* is already established, is deforestation associated with increased rat lungworm disease incidence? Given the coarse resolution and incomplete coverage of data on rat lungworm disease, our analysis is meant to explore possible mechanisms by which species introductions and deforestation can simultaneously impact human health.

## Materials and Methods

We used R version 3.5.3 to run all statistical analyses (42) and QGIS version 3.8 (43) to extract climate and forest cover variables.

### Trade and Introduction of *A. cantonensis*

We built regression models to examine the role of global trade in the introduction and establishment of *A. cantonensis* into countries, while controlling for climatic and biogeographic variables that can affect the successful establishment of the parasite. We focused on the trade of snails, live plants, vegetables, and fruits because adult snails are often deliberately or accidentally introduced through these trade products (44). Rat introductions are not typically associated with any specific trade product; thus, we assumed rats to also be accidentally introduced through the trade of any of these commodities. We used these models to estimate the probability of introduction of *A. cantonensis* into countries where the parasite has not been reported.

#### Reports of *A. cantonensis*

We used the November 2020 reports of the Global Infectious Diseases and Epidemiology Online Network (GIDEON) (38) to classify countries where *A. cantonensis* is present if autochthonous transmission of the parasite (i.e., the parasite was locally acquired) in animals or humans had been reported, and countries where *A. cantonensis* is absent if neither the parasite nor cases of the disease had been reported, although apparent absence may be due to the lack of detection. While most information sources in the GIDEON database are peer-reviewed publications, some are based on ProMED (https://promedmail.org/), which reports new and ongoing outbreaks that may not have yet been peer-reviewed. The GIDEON database has been used widely for studies analyzing global disease patterns [e.g., (45–47)].

#### Trade

We used the BACI International Trade database (48) to obtain the average quantities (in metric tons) of snails, live plants, vegetables, and fruits traded by country during the years 2000, 2005, 2010, and 2015. This database combines snails that are traded alive, fresh, chilled, frozen, dried, salted, or in brine, and the database does not specify the snail species being traded, but only live and fresh snails can serve as vectors for *A. cantonensis*. Because trade quantities have been shown to positively correlate with species introductions (49), for each trade product we selected countries for which exporting quantities fell at or above the 90th percentile of total quantities. We then grouped these exporters depending on their *A. cantonensis* status (i.e., present and absent) by summing the average quantities of exported products (Supplementary Material 1).

#### Climatic Variables

Temperature and precipitation have been shown to be good predictors of habitat suitability for *A. cantonensis*, with regions with high precipitation and temperatures being more suitable (50). We obtained monthly maximum temperature and monthly precipitation gridded data at a 4 km spatial resolution from TerraClimate (51) for the years 2000, 2005, 2010, and 2015 and calculated the mean maximum temperature and mean precipitation values across years. Autochthonous cases of rat lungworm disease have been reported in different regions of the United States and China (Supplementary Material 2), and given the large area and variability in temperature and precipitation values within these countries, we only considered the temperature and precipitation values for the regions where the parasite or disease had been reported.

#### Island Status

We created a dummy variable classifying countries as islands (1) or non-islands (0) to control for a potential island effect driven by the dependence of several islands' economy on global trade (39).

#### Statistical Analysis

We first tested for spatial autocorrelation in the distribution of *A. cantonensis* reports across countries using the joint count statistical test (*spdep* package) and found no spatial autocorrelation (join count test *p* = 0.8). We then examined the effect of trade from countries where *A. cantonensis* is present on the likelihood of presence in other countries, while controlling for climate, island status, and product quantities exported by countries where *A. cantonensis* is absent. We tested the effect of trade on likelihood of presence for each product independently to avoid overfitting a single model with the variables from the four trade products. For each trade product, we fitted the presence of *A. cantonensis* across countries using generalized linear models with binomial distributions. Trade and climatic predictor variables were standardized (mean = 0 and standard deviation = 1). For each trade product, we used the Akaike Information Criterion (AIC) approach to compare and select among a set of four models that included variations of the full model with trade, climate, and island status variables (Supplementary Material 3). We selected final models based on the lowest AIC score corrected for small sample size (AICc) and the highest AICc weight, which ranges from 0 to 1 and represents the likelihood that a given model is the best among the candidate set. We estimated the average accuracy of each best-fitting regression model using leave-one-out cross-validation (LOOCV) and used the most accurate model to estimate the probability of *A. cantonensis* introduction (*predict* function) into countries where *A. cantonensis* is currently absent.

### Deforestation and Rat Lungworm Disease

We examined the effect of deforestation on rat lungworm disease incidence at regional scales, while controlling for climatic variables that can affect presence and abundance of the parasite. We focused on Hawaii and Thailand, where *A. cantonensis* is present and where epidemiological information for rat lungworm disease was available at a spatial scale.

#### Rat Lungworm Disease

The number of rat lungworm human cases for the state of Hawaii were available at the ZIP Code Tabulation Area (ZCTA) level for every year between 2007 and 2017 (52) (Supplementary Material 4). For Thailand, the available number of human cases were reported at the province level only for the year 2017 (53) (Supplementary Material 4). For both locations, we estimated incidence by dividing the annual number of cases by the population size at the corresponding administrative level (i.e., ZCTA or province). We obtained population size for Hawaii for the year 2015 from the US Census Bureau (54) and for Thailand from the Humanitarian Data Exchange (55).

#### Deforestation

We used a high-spatial-resolution (30-m) dataset that quantifies global forest cover loss by year (10). We used cumulative forest cover loss because ecological factors that may lead to *A. cantonensis* enzootic or zoonotic transmission, such as rat and snail establishment and invasion, may exhibit a time lag in their responses to deforestation. For Hawaii, disease incidence was available for each year between 2007 and 2017; thus, we extracted the fraction of forest cover lost in each ZCTA for each year and calculated the annual cumulative forest cover loss by summing the previous year(s) forest cover lost to each year (Supplementary Material 4). For Thailand, disease incidence was only available for 1 year, but we extracted forest cover loss at the province level for the years 2007 through 2017 to calculate the cumulative forest cover lost during that period (Supplementary Material 4).

#### Climatic Variables

We calculated the mean maximum temperature and mean precipitation from monthly maximum temperature, and monthly precipitation gridded data at a 4 km spatial resolution (51).

#### Statistical Analysis

We tested for spatial autocorrelation in the distribution of rat lungworm disease incidence in both regions by calculating Moran's I (*spdep* package) using the Monte Carlo method with 1,000 simulations and found no spatial autocorrelation in Hawaii (Moran's I *p* = 0.35) nor Thailand (Moran's I *p* = 0.59). A large proportion of ZCTA in Hawaii and provinces in Thailand had zero reported rat lungworm disease cases, resulting in overdispersion of rat lungworm disease incidence. Thus, we used a hurdle model approach to examine the effect of deforestation on rat lungworm disease by fitting occurrence of rat lungworm cases (presence–absence) to the full datasets, and incidence to datasets with reported cases only. For Hawaii, we fitted rat lungworm disease with generalized linear mixed models (occurrence = binomial family, logit link; incidence = Gamma family, log link) and included a random intercept by ZCTA and year. For Thailand, we fitted rat lungworm disease with generalized linear models (occurrence = binomial family, logit link; incidence = Gamma family, log link). For both regions, we log-transformed rat lungworm disease incidence after adding one and standardized the predictor variables (mean = 0 and standard deviation = 1). For each region, we used AIC to compare and select among a set of three models that included the full model with forest cover loss and climate variables (Supplementary Material 5). We selected final models based on the lowest AICc score and the highest AICc weight.

## Results

### Trade and Introduction of *A. cantonensis*

**Figure 1:**
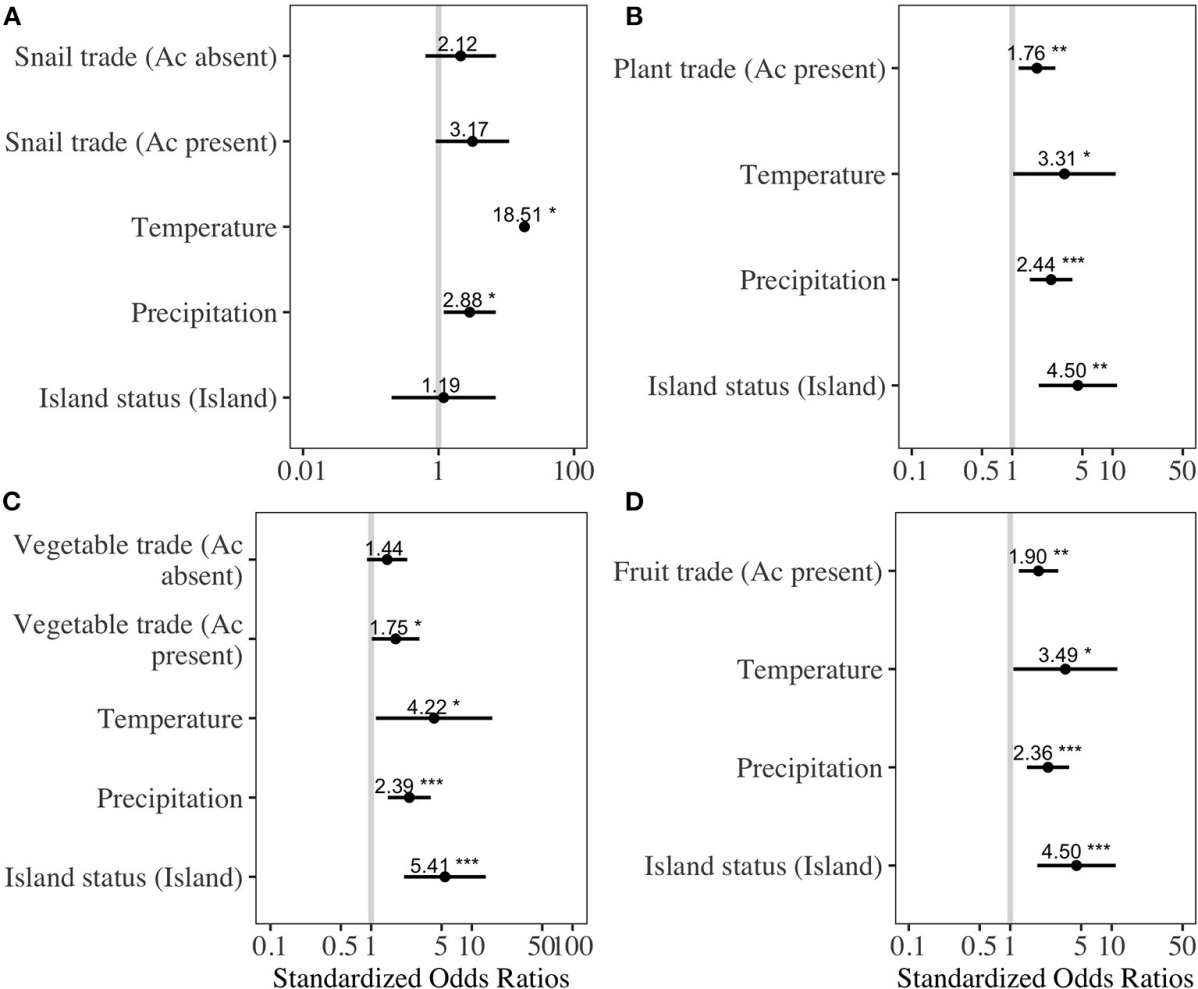
Best-fitting regression models for the presence of *Angiostrongylus cantonensis* at the country level. To avoid overfitting, we fit separate models based on average annual traded quantities of **(A)** snails, **(B)** live plants, **(C)** vegetables, and **(D)** fruits. *Ac present* refers to product quantities exported from countries where *A. cantonensis* is present, and *Ac absent* refers to exports from countries where the parasite is absent. Dots indicate the standardized model estimates with bars representing 95% confidence intervals; vertical line is the line of null effect. Asterisks indicate the level of statistical significance (* = 0.05, ** = 0.01, *** = 0.001).

As of November 2020, 49 countries had reported the presence of either autochthonous *A. cantonensis* human infections or animal infections (Supplementary Material 6) (38). The best-fit models explaining the presence of *A. cantonensis* contained either all variables (Supplementary Material 3: snail trade model and vegetable trade model) or included all variables except trade quantities from countries where *A. cantonensis* was absent. The odds of parasite presence significantly increased with average annual trade from countries where *A. cantonensis* was present for the following products: live plants (OR = 1.7, *p* = 0.01, LOOCV= 83%), vegetables (OR = 2.3, *p* = 0.04, LOOCV= 83.6%), and fruits (OR = 1.98, *p* = 0.003, LOOCV = 82.1%) (Figure 1). Odds of parasite presence were not significantly associated with the trade of snails from countries where *A. cantonensis* was present (Figure 1). Specifically, the effect of trade on the presence of *A. cantonensis* in importing countries was significantly positive only for horticultural products traded with countries where the parasite is present (Figure 1).

**Figure 2:**
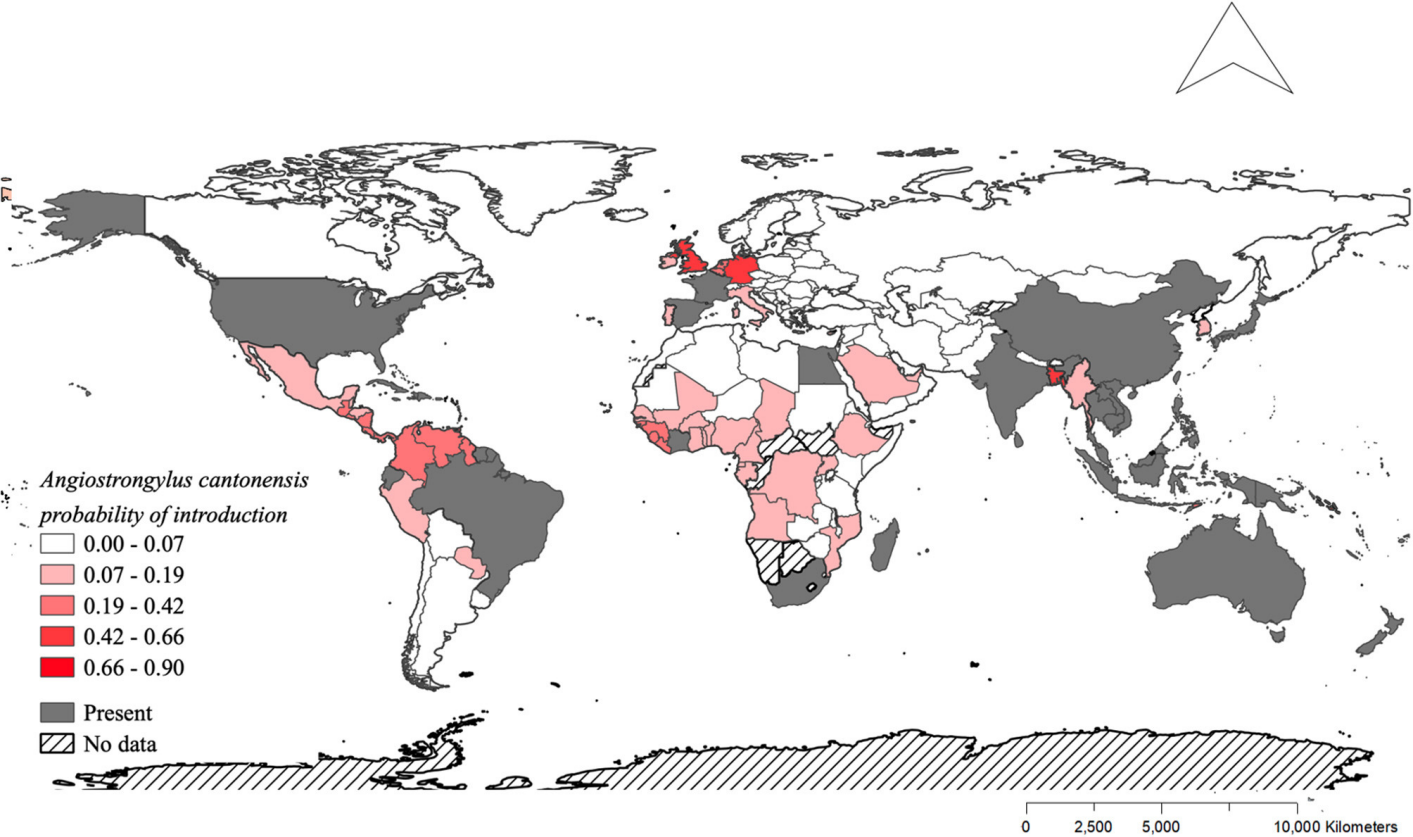
Distribution of *Angiostrongylus cantonensis* and predicted probabilities of future introduction. Shades of red indicate the probabilities of future introduction based on the fitted model for exported vegetables (Figure 1C). Gray indicates the countries where *A. cantonensis* is already present, and hashed pattern indicates the countries that were not included in the model due to the absence of data.

The model using traded vegetables had the highest average predictive accuracy (LOOCV = 83.6%). Based on this model, the probability of *A. cantonensis* introduction is predicted to be ≥50% for 13 of 149 countries with available trade information (Figures 1, 2, Table 1, Supplementary Material 6), including the Solomon Islands (89.7%), the United Kingdom (65.5%), Wallis and Futuna Islands (65.5%), Kiribati (64.5%), Seychelles (62.1%), and Germany (59.9%) (Table 1).

**Table 1:** Countries with ≥50% predicted probability of *Angiostrongylus cantonensis* introduction based on the best-fitting model for quantities of exported vegetables.

**Country**	**Vegetable trade: *A. cantonensis* present (thousand metric tons)**	**Vegetable trade: *A. cantonensis* absent (thousand metric tons)**	**Mean maximum temperature (Celsius)**	**Mean precipitation (inches)**	**Island**	**Predicted probability estimates**
Solomon Is.	0.66	0	29.67	336.41	Yes	0.90
United Kingdom	1,219.35	1,156.58	12.37	95.50	Yes	0.65
Wallis and Futuna Is.	0.08	0	30.02	208.34	Yes	0.65
Kiribati	0.21	0.01	31.33	193.19	Yes	0.64
Seychelles	2.94	1.89	29.49	200.48	Yes	0.62
Germany	1,491.80	2,505.66	13.43	65.34	No	0.60
Saint Lucia	3.06	2.56	30.42	184.32	Yes	0.60
Nauru	0	30.87	178.62	Yes	0.59
St. Vincent and Grenadines	0.65	1.50	29.57	182.66	Yes	0.57
Maldives	15.63	0.57	30.79	166.81	Yes	0.56
Trinidad and Tobago	17.35	42.08	30.49	166.05	Yes	0.56
Dominica	0.379	0.91	28.97	169.92	Yes	0.51
Barbados	5.98	10.18	31.10	146.30	Yes	0.51

*Absolute values of trade and climatic variables are shown and represent the average annual values. Vegetable trade is grouped by A. cantonensis status of exporting country (present and absent)*.

### Deforestation and Rat Lungworm Disease

Within two regions where *A. cantonensis* has been introduced, Hawaii and Thailand, we investigated how rat lungworm disease occurrence (i.e., presence of cases) and incidence (i.e., number of cases in a population per unit time) related to climate and forest cover loss. In Hawaii, seventy-nine cases were reported during the years 2007 through 2017 with sufficient information to assign to a ZCTA (52). These cases were reported in 21 of 94 ZCTA. Rat lungworm disease occurrence was best explained by the model including only climatic variables, with odds of occurrence significantly associated with increasing mean precipitation (Table 2, Supplementary Material 5). Rat lungworm disease incidence was best explained by the full model, with incidence significantly associated with cumulative forest cover loss (Table 2, Supplementary Material 5).

**Table 2:** Best-fitting hurdle-based models explaining the effect of forest cover loss on rat lungworm disease occurrence and incidence in Hawaii and Thailand.

	**Disease occurrence**			**Disease incidence**		
**Predictors**	**Odds ratios**	**95% CI**	***p*-value**	**Estimates**	**95% CI**	***p*-value**
**HAWAII**						
Cumulative forest cover loss	†	†	†	1.10	1.04–1.16	<0.001
Mean precipitation	2.44	1.43–4.15	<0.01	1.10	0.97–1.26	0.14
Maximum temperature	0.64	0.33–1.24	0.19	0.80	0.64–1.01	0.06
**THAILAND**						
Cumulative forest cover loss	†	†	†	4.13	0.9–17.30	0.05
Mean precipitation	†	†	†	0.03	0.00–0.47	0.02
Maximum temperature	†	†	†	0.31	0.16–0.58	<0.01

*Occurrence of rat lungworm cases (presence and absence) was fitted to the full datasets, and incidence (number of cases in a population per unit time) was fitted to datasets with reported cases only. Model selection was based on model with lowest AICc. Indicates variable was not included in the final model†*.

In Thailand, 181 cases were reported in 18 of 77 provinces during 2017 (53). Rat lungworm disease occurrence was best explained by the null model (Table 2, Supplementary Material 5), and disease incidence was best explained by forest cover loss, mean annual precipitation, and mean maximum temperature, in which the effect of forest cover loss was marginally significant (Table 2, Supplementary Material 5).

## Discussion

Our combined findings suggest that global trade increases the likelihood of introducing infected hosts into new countries, and regional deforestation can amplify their human health impacts. We found the trade of horticultural products to be an important driver in the spread of the parasite across the globe (Figure 1). Using the trade model with the highest predictive accuracy, we found that most countries at high risk of future *A. cantonensis* introduction are islands (Table 1). At regional levels, deforestation appeared to play an important role in the per-capita risk of *A. cantonensis* exposure in Hawaii and only marginally in Thailand (Table 2). Our study provides a coarse view of the associations between the introduction of zoonotic reservoirs, deforestation, and risk of *A. cantonensis* exposure. Better understanding at finer temporal and spatial scales how these two widespread and overlapping sources of environmental change affect human health can inform international biosecurity protocols, invasive species management, and land-use policies.

Our results suggest the trade of vegetables, fruits, and live plants facilitates the introduction of *A. cantonensis* into countries where climatic conditions are favorable for parasite development (Figure 1). Specifically, trade from large exporting countries where *A. cantonensis* is present appears to constitute a source of propagule pressure of infected hosts into new regions. Importantly, the probability that introduced hosts are indeed infected is largely dependent on the prevalence of the parasite in host populations from the exporting country, not simply its presence (56). Thus, a more accurate estimation of the relative risk of introduction would require estimates of *A. cantonensis* prevalence in rats or snails from exporting countries, which are currently available for few of the countries included in our analysis (57). Furthermore, other factors such as within-country variability in habitat suitability for hosts, as well as proximity to countries where the parasite is present and biophysical barriers to host movement, could help determine the role of trade in *A. cantonensis* introduction.

The trade of snails appeared to have no effect on the odds of parasite introduction; however, the information used in the model combined all snail products, of which only a subset may carry the infective stage of *A. cantonensis* (i.e., live and fresh snails). Thus, to better assess the role of this commodity on the introduction of *A. cantonensis* would require information on snail quantities to be segregated by type. Lastly, while we based our analysis on trade products through which snails are commonly introduced, the risk of parasite introduction through rats *via* other trade products should not be disregarded, as the transboundary movement of rats is likely not restricted to horticultural products.

Island nations appear to be at greater risk of *A. cantonensis* introduction than mainland countries (Figure 1, Table 1). The size, isolation, and limited resource diversity of many islands worldwide lead to substantial dependence on global imports (39–41), making them more vulnerable to species introductions. This, combined with many islands' lack of native predators and competitors, creates ideal conditions for introduced species like rats and several important *A. cantonensis* snail hosts (e.g., giant African snails and semi-slugs) (58, 59). Due to their impacts on island ecosystems and endemic species, introduced species are often targeted for eradication (60), and these conservation efforts can simultaneously result in public health benefits (61). For example, the eradication of rats is currently feasible in 39 human-inhabited islands, including Curieuse Island in Seychelles (62), where the probability of *A. cantonensis* introduction is high (Table 2). Eradicating rats, the definitive host for *A. cantonensis*, from Curieuse Island could thus eliminate the local risk of *A. cantonensis* introduction and exposure in humans and animals. Furthermore, considering the potential role of global trade in the introduction of zoonotic hosts, risk to islands could be reduced through enhanced international cooperation toward stricter biosecurity protocols [e.g., (9)].

Our results indicate that the per-capita risk of exposure to *A. cantonensis* increased with deforestation in Hawaii and the effect was marginally significant in Thailand (Table 2). Specifically, deforestation in Hawaii had a positive effect on rat lungworm disease incidence but not on occurrence, suggesting deforestation may affect the rate at which humans encounter infected hosts (i.e., incidence), but may not play an important role in the establishment of the zoonotic cycle in a given location (i.e., occurrence). Thus, deforestation may be creating conditions conducive to *A. cantonensis* transmission by increasing contact between people, rats, and snails, particularly snail species like the semi-slug (*Parmarion cf. martensi*) that thrive in anthropic environments and whose introduction to Hawaii has been associated with a concurrent rise in rat lungworm disease cases (27). Exposure to infected snails may also be affected by socioeconomic factors, such as the presence of unregulated rainwater catchment where snails have been shown to drown and release infective *A. cantonensis* larvae [e.g., (63)], or by cultural factors, such as the use of snails in local foods [e.g., (64)]. Lastly, other studies have used different environmental factors such as wind speed, as predictors of rat lungworm disease in humans [e.g., (65)]. To better understand the mechanisms by which deforestation increases the incidence of rat lungworm disease would require epidemiological information, risk factor analyses that include environmental, socioeconomic, and cultural factors, as well as estimates of *A. cantonensis* prevalence in host populations compared across gradients of deforestation.

## Conclusion

The coarse resolution of our analyses provides a preliminary understanding of the mechanisms by which species introductions and deforestation can simultaneously increase the risk of exposure to zoonotic pathogens such as *A. cantonensis*. Preventing the introduction and spread of non-native species, which is a common approach to protect biodiversity, can also reduce the emergence of zoonotic pathogens and exposure to people. The implementation of strict biosecurity protocols at ports of entry can help reduce the rate of species introductions (9, 66). Populations of zoonotic reservoirs can be directly managed to reduce local exposure to pathogens [e.g., (61)] or indirectly through targeted ecosystem management approaches aimed at reducing suitable habitat for invasive species. For example, invasive snail and rat populations can be controlled by restoring wetlands with species that are not palatable for invasive snails (67), or through urban planning that incorporates proper disposal of solid waste and other resources that could otherwise subsidize zoonotic reservoirs like rats (68). Our study explored how introduced species and deforestation can have amplifying impacts on human health. With increasing trends in globalization and land-use conversions, measures aimed at sustainably managing economic activities and land-use patterns are likely to be important in reducing the health burden from emerging zoonotic diseases.

## Code Availability

The data and code for analyses of this study are available through doi: 10.6084/m9.figshare.12298910.

## Data Availability Statement

The original contributions presented in the study are included in the article/Supplementary Material, further inquiries can be directed to the corresponding author/s.

## Author Contributions

LW: study design, literature search, data collection, data analysis, data interpretation, figures, and writing. TR: study design, data analysis, data interpretation, and writing. Both authors contributed to the article and approved the submitted version.

## Funding

LW was supported by a Gund Institute Postdoctoral Fellowship at University of Vermont.

## Conflict of Interest

The authors declare that the research was conducted in the absence of any commercial or financial relationships that could be construed as a potential conflict of interest.

## Publisher's Note

All claims expressed in this article are solely those of the authors and do not necessarily represent those of their affiliated organizations, or those of the publisher, the editors and the reviewers. Any product that may be evaluated in this article, or claim that may be made by its manufacturer, is not guaranteed or endorsed by the publisher.
