# Supplementary material for: Trade and Deforestation Predict Rat Lungworm Disease, an Invasive-Driven Zoonosis, at Global and Regional Scales
Source: Front Public Health. 2021 Sep 9;9:680986. doi: 10.3389/fpubh.2021.680986 (PMC8458565; doi:10.3389/fpubh.2021.680986)
Supplement: Supplementary file 1 [file Data_Sheet_1.zip › Tables 2, 3, 5.DOCX]

**SI 2.** Regions within the United States of America and China where autochthonous cases of rat lungworm disease have been reported as of November 2020.

| **United States of America** | **China** |
| --- | --- |
| - Puerto Rico - Florida - Louisiana - Tennessee - Texas - Hawaii | - Beijing Municipality - Fujian Province - Guangdong Province - Guangxi Zhuang Autonomous Region - Guizhou Province - Hainan Province - Hunan Province - Yunnan Province - Zhejiang Province |

**SI 3.** Trade model selection using Akaike Information Criterion corrected for small sample size (AICc). For each trade product presence of *Angiostrongylus cantonensis* (AcPresence) was fit to a set of candidate models, represented in each row. The best fit model is highlighted in bold and was selected based on the lowest AICc score and highest AICc weight. TradeAcPresent represents trade quantities from countries where *A. cantonensis* was present, and TradeAcAbsent represents trade quantities from countries where *A. cantonensis* was absent.

| **Model** | **AICc** | **AICc weight** |
| --- | --- | --- |
| AcPresence ~ TradeAcPresent + TradeAcAbsent + Temperature + Precipitation + Island status | **Snail: 56.49**  Live plants: 154.71  **Vegetables: 152.05**  Fruits: 160.58 | **Snail: 0.78**  Live plants: 0.24  **Vegetables: 0.64**  Fruits: 0.26 |
| AcPresence ~ TradeAcPresent + Temperature + Precipitation + Island status | Snail: 59.32  **Live plants: 152.63**  Vegetables: 153.23  **Fruits: 158.52** | Snail: 0.19  **Live plants: 0.69**  Vegetables: 0.36  **Fruits: 0.73** |
| AcPresence ~ Temperature + Precipitation + Island status | Snail: 62.86  Live plants: 157.14  Vegetables: 166.60  Fruits: 166.60 | Snail: 0.03  Live plants: 0.07  Vegetables: 0  Fruits: 0.01 |
| AcPresence ~ 1 | Snail: 85.82  Live plants: 204.21  Vegetables: 215.08  Fruits: 215.08 | Snail: 0  Live plants: 0  Vegetables: 0  Fruits: 0 |

**SI 5.** Rat lungworm disease hurdle model selection using Akaike Information Criterion corrected for small sample size (AICc) for Hawaii and Thailand. Rat lungworm disease occurrence and incidence were fit to a set of candidate models, represented in each row. For the Hawaii models, random effects for year and ZCTA were retained in all model variations. The best fitting models are highlighted in bold and were selected based on the lowest AICc score and highest AICc weight.

| Model | AICc | AICc weight |
| --- | --- | --- |
| Occurrence |  |  |
| Occurrence ~ Cum Forest Loss + Precipitation + Temperature | Hawaii: 271.92  Thailand: 89.10 | Hawaii: 0.38  Thailand: 0.12 |
| Occurrence ~ Precipitation + Temperature | **Hawaii: 271**  Thailand: 87.63 | **Hawaii: 0.60**  Thailand: 0.25 |
| Occurrence ~ 1 | Hawaii: 278.2  **Thailand: 85.80** | Hawaii: 0.02  **Thailand: 0.63** |
| Incidence |  |  |
| Incidence ~ Cum Forest Loss + Precipitation + Temperature | **Hawaii: 85.31**  **Thailand: -30.20** | **Hawaii: 0.94**  **Thailand: 0.76** |
| Incidence ~ Precipitation + Temperature | Hawaii: 93.84  Thailand: -27.89 | Hawaii: 0.01  Thailand: 0.24 |
| Incidence ~ 1 | Hawaii: 91.52  Thailand: -20.31 | Hawaii: 0.04  Thailand: 0.01 |
